# The Prolactin Inducible Protein Modulates Antitumor Immune Responses and Metastasis in a Mouse Model of Triple Negative Breast Cancer

**DOI:** 10.3389/fonc.2021.639859

**Published:** 2021-03-12

**Authors:** Chidalu A. Edechi, Nnamdi M. Ikeogu, Gloria N. Akaluka, Lucas E. L. Terceiro, Mikayla Machado, Enitan S. Salako, Aida F. Barazandeh, Sam K. P. Kung, Jude E. Uzonna, Yvonne Myal

**Affiliations:** ^1^Department of Pathology, Max Rady College of Medicine, University of Manitoba, Winnipeg, MB, Canada; ^2^Department of Immunology, Max Rady College of Medicine, University of Manitoba, Winnipeg, MB, Canada; ^3^Department of Physiology and Pathophysiology, Max Rady College of Medicine, University of Manitoba, Winnipeg, MB, Canada; ^4^Research Institute in Oncology and Hematology (RIOH), CancerCare Manitoba, Winnipeg, MB, Canada

**Keywords:** breast cancer, prolactin inducible protein, lung metastasis, mouse models, immune response

## Abstract

The prolactin inducible protein (PIP) is expressed to varying degrees in more than 90% of breast cancers (BCs). Although high levels of PIP expression in BC has been shown to correlate with better prognosis and patient response to chemotherapy, some studies suggest that PIP may also play a role in metastasis. Here, we investigated the role of PIP in BC using the well-established 4T1 and E0771 mouse BC cell lines. Stable expression of PIP in both cell lines did not significantly alter their proliferation, migration, and response to anticancer drugs *in vitro* compared to empty vector control. To assess the effect of PIP expression on breast tumorigenesis *in vivo*, the 4T1 syngeneic transplantable mouse model was utilized. In immunocompetent syngeneic BALB/c mice, PIP-expressing 4T1 primary tumors displayed delayed tumor onset and reduced tumor growth, and this was associated with higher percentages of natural killer cells and reduced percentages of type 2 T-helper cells in the tumor environment. The delayed tumor onset and growth were abrogated in immunodeficient mice, suggesting that PIP-mediated modulation of primary tumor growth involves an intact immune system. Paradoxically, we also observed that PIP expression was associated with a higher number of 4T1 colonies in the lungs in both the immunocompetent and immunodeficient mice. Gene expression analysis of PIP-expressing 4T1 cells (4T1-PIP) revealed that genes associated with tumor metastasis such as CCL7, MMP3 and MMP13, were significantly upregulated in 4T1-PIP cells when compared to the empty vector control (4T1-EV) cells. Collectively, these studies strongly suggest that PIP may possess a double-edge sword effect in BC, enhancing both antitumor immunity as well as metastasis.

## Introduction

Breast cancer (BC) remains the most common cancer among females, affecting over 2 million women globally (1). Both genetic and epigenetic alterations in normal breast cells can result in their transformation into BC (2). Currently, more than 10 molecular subtypes have been identified but the most common are: the luminal A and luminal B, human epidermal growth factor receptor 2 expressing, and the triple-negative BCs, TNBC (3). These subtypes generally display significantly different biological characteristics underscoring the heterogenous nature of the disease.

In addition to cancer cells, the tumor microenvironment is composed of a wide variety of other cell types, including cancer associated fibroblasts and immune cells, which greatly impact tumor progression (2). In particular, the immune system is known to play a pivotal role in cancer development (4). Through rigorous immunosurveillance, the host immune system often eliminates a developing tumor before the tumor is established (5). Various immune cells such as CD8^+^ T cells, CD4^+^ type 1 T-helper (Th1) cells, natural killer (NK) cells, classically activated (M1) macrophages and mature dendritic cells (DCs) contribute to tumor elimination and thus are considered antitumorigenic. In contrast, immature DCs, CD4^+^ type 2 T-helper (Th2) cells, CD4^+^ T regulatory cells, myeloid-derived suppressor cells, and alternatively activated (M2) macrophages are pro-tumorigenic (6–9). Although BC was not previously considered an immunogenic cancer, accumulating evidence, including the presence of immune cells in some BCs, suggests that these immune cells have prognostic and therapeutic relevance and significance. Indeed, a recent report showed that patients who have tumors with a Th2 profile, have a worse prognosis than patients with a Th1 or CD8^+^ T cell profile (10).

Although there has been significant progress in the treatment of BC, metastasis still poses a serious challenge and accounts for over 90% of BC deaths (11). During metastasis, cancer cells may undergo epithelial to mesenchymal transition (12) and also release matrix metalloproteinases which degrade the basement membrane proteins. These processes collectively facilitate the escape of the cancer cells from the primary tumor site into the circulation and then to distal organs where they embark (13). At the metastatic site, these disseminated cancer cells adapt to their new environment and interact with the surrounding stromal cells to promote their survival (12). The common metastatic sites for BC are the lymph nodes, bone, lung, liver, and brain (14). Recent estimates show that about 6 to 10% of women present with stage 4 metastatic BC at the time of diagnosis (11). A better understanding of the mechanisms underlying the metastatic process is a critically important step for the development of effective therapies that will further reduce the mortality rate for patients with metastatic BC.

The prolactin inducible protein (PIP) is a 15–17 kDa glycoprotein, initially identified as a highly secreted protein in T47D BC cells *in vitro* following treatment with prolactin and androgens (15). PIP is expressed in abundance by BC cells but is generally found to be low or absent in normal breast cells (16–19). The PIP gene which is 7 kb long and has four exons is regulated by hormones such as prolactin and androgens (20–22). It has been determined that greater than 90% of BCs express PIP (also known as gross cystic disease protein-15) to some degree (23). As a result, PIP is now frequently used in the clinic as one of the few key biomarkers to confirm if an unknown metastatic cancer has originated from the breast (18, 19, 24). A correlation between ER expression and PIP has recently been reported. The highest levels of PIP mRNA expression were identified in the luminal A molecular subtype (ER+, PR+) of BC. Lower levels of PIP expression were observed in other molecular BC subtypes (25, 26). More recently, PIP expression in human BC patients was shown to be associated with better prognosis and response to chemotherapy (27–30). Although the function of PIP in BC has not yet been delineated, early studies from our laboratory (31–35) and others (36, 37) point to an immunoregulatory role in both innate and adaptive immune responses. As well, PIP is found at sites considered to be the first line of defense against invading organisms such as the eyes (lacrimal gland), skin (sweat glands), ears and mouth (salivary glands) (38, 39). Additionally, our laboratory has previously demonstrated in mice that PIP plays a direct role in the aggregation of oral bacteria, thereby inhibiting their spread, a mechanism known to be important in innate host defense (40, 41).

Further work using PIP knock out (KO) mice that we generated (34) demonstrated that PIP KO mice exhibited numerous alterations in their oral flora (40) and displayed abnormalities in key lymphoid organs as well as in the Eustachian tube of the ear (34, 42). Gene expression analysis revealed that deficiency of PIP was associated with differentially expressed genes involved in the immune response as well as in cancer pathogenesis (34). Importantly, using these PIP KO mice, we identified a direct and critical role for PIP in the adaptive immune response (32–34). We demonstrated that CD4^+^ Th1 differentiation was impaired in PIP KO mice (32), revealing for the first time, that PIP is critical for optimal induction of cell-mediated immunity. Furthermore, PIP deficiency was also found to be associated with impaired intracellular signaling events in macrophages and DCs leading to decreased production of pro-inflammatory cytokines (33). These findings, in addition to the expression of PIP in most BCs led us to hypothesize a role for PIP in antitumor immunity during BC development and progression.

In this study, we investigated the effect of PIP in BC using the well-established mouse BC cell lines, 4T1 and E0771 cells. We generated PIP-expressing 4T1 and E0771 cells and evaluated the effect of PIP on these cells *in vitro* using several functional assays. We also performed *in vivo* studies using the 4T1 syngeneic transplantable mouse model to examine the effect of PIP on 4T1 breast tumor development, immune response and metastasis. Our results suggest that PIP may play a dual role in modulating antitumor immunity as well as BC metastasis.

## Materials and Methods

### Cell Lines

The 4T1 and E0771 mouse BC cell lines were purchased from the American Type Culture Collection (ATCC, Manassas, VA) and CH3 biosystems (Amherst, NY) respectively without further characterization and were screened for mycoplasma before storage and prior to use in experiments. The cells were cultured at 37°C and 5% CO_2_ in complete medium consisting of high-glucose (4.5 g/L) Dulbecco’s Modified Eagle’s Medium (DMEM; Hyclone Laboratories Inc., Logan, UT) containing 10 μg/ml bovine insulin (Sigma-Aldrich Canada Co., Oakville, ON, Canada), 2 mM glutamine, 10% fetal bovine serum (FBS), 50 μg/ml streptomycin and 50 U/ml penicillin (all obtained from Hyclone Laboratories Inc.). The cells were passaged every 2–3 days at 85–100% confluency.

### Generation of 4T1 and E0771 Cells That Stably Express Prolactin Inducible Protein

Lentiviral constructs for PIP and the empty vector controls were designed in our laboratory and generated by Vector Builder (Chicago, IL). In the vector design, the mouse PIP coding region was under the control of the elongation factor 1 alpha (EF1a) promoter. Enhanced green fluorescent protein (GFP) was used as the reporter and was placed under the control of the cytomegalovirus (CMV) promoter. A similar vector design without the PIP coding region was used as the empty vector (EV) control. The PIP vector and the empty vector constructs were packaged into lentiviral particles (Vector Builder) for transduction/infection of the cell lines. Briefly, 5 × 10^4^ 4T1 or E0771 cells/well were cultured overnight in 24 well tissue culture plates. The following day, the cells were then incubated with complete DMEM containing lentivirus encoding either PIP or EV (viral concentration of 50 multiplicity of infection, MOI) and polybrene (8 µg/ml) at 37°C overnight. Following incubation, the virus was discarded and replaced with fresh complete DMEM. The cells were visualized by fluorescence microscopy 48–72 h later to identify successfully transduced cells. As well, transduced cells were analyzed by flow cytometry to determine the percentage of GFP expressing cells. The cells were acquired on flow activated cell sorter (FACS) Canto II (BD biosciences, San Diego, CA), gated on live cells and then GFP expressing cells. Subsequently, GFP expressing cancer cells were sorted by flow cytometry using the FACS Aria III (BD biosciences). The presence of PIP in transduced 4T1 and E0771 cells was confirmed by Western blot analysis.

### Western Blot Analysis

The cells were grown to confluency, rinsed with 1× phosphate buffered saline (PBS) and lysed using radioimmunoprecipitation assay (RIPA) buffer (Thermo Scientific, Rockford, IL) containing protease inhibitor (Roche Diagnostics, Laval, QC, Canada). Protein concentration in the lysates was quantified by the Bicinchoninic acid (BCA) assay (Pierce Biotechnology, Rockford, IL), according to manufacturer’s instructions. The samples were mixed with reducing agent (dithiothreitol) and loading dye (both from Life Technologies, Carlsbad, CA) and heated to 70°C for 10 min. Then, samples were electrophoresed in Bolt™ 4–12% Bis-Tris Plus protein gels (Life Technologies). Proteins were then transferred to nitrocellulose membrane using the Pierce Power Blot Cassette (Thermo Scientific) and blocked with 5% non-fat milk in Tris-buffered saline containing 0.1% Tween-20 (TBS-T) for 1 h. The membrane was then incubated with rabbit anti-mouse mouse PIP antibody (1:2,500 diluted in blocking solution; Alpha diagnostics, San Antonio, TX) overnight at 4°C. Thereafter, the membrane was washed with TBS-T, three times for 10 min and incubated with HRP-conjugated goat anti-rabbit secondary antibody (1:10,000, Bio-Rad, Mississauga, ON, Canada) at room temperature for 1 h. The secondary antibodies were washed off with TBS-T (10 min, three times) and developed using the Immobilon enhanced chemiluminescence (ECL) kit (Millipore, Billerica, MA). Signal was acquired using the C-digit blot scanner (Li-cor, Lincoln, NE).

### Cell Counting by Trypan Blue Exclusion

The 4T1 or E0771 cells (WT, EV, and PIP) were grown in triplicates in 12-well plates at a concentration of 5 × 10^4^ cells/well. The cells were detached using 0.05% trypsin (Hyclone Laboratories Inc.), mixed with trypan blue (1:1) and counted daily for 4 days using a TC-10 cell counter (Bio-Rad Laboratories Inc).

### XTT Assay

XTT cell proliferation assay kit (ATCC) was used to measure cell metabolic activity according to the manufacturer’s instructions. Briefly, 5 × 10^4^ cells/well (24-well tissue culture plate) were grown in complete DMEM medium for 24 h or 4 days. Thereafter, XTT reagents were added to the cell culture and incubated for 2–4 h after which an orange coloration was observed. Absorbance was measured at wavelengths of 475 nm and 660 nm using a Spectra Max 190 (Molecular Devices, San Jose, CA).

### Drug Sensitivity Assays

The cells (5 × 10^3^/well) were grown overnight in 96-well plates and treated with dimethyl sulfoxide (DMSO, vehicle control), doxorubicin (0.5 μM), tamoxifen (10 μM), etoposide (50 μM) or cisplatin (20 μM) for 48 h (all from Sigma-Aldrich). Cell viability was assessed using the XTT assay as described above. Results were normalized to DMSO control.

### Wound Healing Assay

Cells were seeded in 12-well plates in triplicates and grown to confluency in complete DMEM medium. A wound (scratch) was created through the cell monolayer using a pipet tip and a fresh culture medium was added. The images of the cells were captured at 0- and 6-h post-wounding using the 10× objective of the microscope attached to a camera (ScopePhoto 3.0, ScopeTek DCM130 microscope camera). Three images were captured per well and analyzed using the Image J program (National Institutes of Health). Migration areas in pixels were measured and plotted.

### Trans-Well Migration Assay

Corning^®^ Costar ^®^ 8 μm Trans-well^®^ plates (Millipore Sigma, Merck, Burlington, MA) was used for this assay. The apical chamber was seeded with 1 × 10^5^ cells in a serum-free culture medium (DMEM). Culture medium supplemented with 30% FBS was added to the bottom (basal) chamber and the cells were incubated at 37°C for 24 h to allow migration through the membrane. Using Q-tips, the inner part of the top chamber/insert was gently cleaned to remove non-migrated cells and the inserts were then immersed in methanol to fix the migrated cells. The cells were stained with crystal violet and photographs of at least five fields/insert were taken. The average number of migrated cells was enumerated.

### Implantation of 4T1 Cells Into Mouse Mammary Fat Pads

Six to 8 weeks old immunocompetent BALB/c mice were obtained from the in-house breeding colony of the University of Manitoba Central Animal Care Services (CACS) and used in this study. As well, the immunodeficient Rag2^−/−^γc^−/−^ double knockout (DKO) BALB/c mice (which lack NK, B and T cells) breeding pairs were originally obtained from Jackson Laboratories (Bar Harbor, ME) and bred in the same facility as immunocompetent BALB/c mice by the CACS. All mice were maintained in a specific pathogen-free environment, exposed to normal light-dark cycles, fed ad-libitum and kept in plastic cages containing wood chip bedding. All experiments were approved by the University of Manitoba Animal Care Committee and conducted according to the guidelines of the Canadian Council on Animal Care (CCAC).

To generate 4T1 tumors, 1 × 10^4^ 4T1-EV or 4T1-PIP cells in 100 μl PBS were injected orthotopically into the 4^th^ mammary fat pad of female wild-type or immunodeficient BALB/c mice as previously described by Pulaski and Ostrand-Rosenberg (43). In each experiment, 3–5 mice were used. Tumor onset and diameter and mouse weight were monitored and recorded every 2 days. The mice were sacrificed at experimental end points, defined by morbidity and loss in weight of more than 10% and/or tumor ulceration. Tumor size was measured in two dimensions using digital calipers and was calculated as follows: Tumor volume (mm^3^)  = (D × d^2^) ÷ 2 (44); where D (mm) = largest diameter of the tumor and d (mm) = the tumor diameter perpendicular to D.

### Tissue Immunophenotyping

At the experimental endpoints, the mice were sacrificed, and breast tumors, spleens and lymph nodes were collected. The tumors were weighed, minced in a Petri dish and digested with collagenase IV (1.5 mg/ml) and DNase 1 (10 μl/ml) in serum-free DMEM for 1 hour at 37°C. Following digestion, the samples were filtered using 40 μm cell strainers and washed twice in complete DMEM to obtain single-cell suspensions. Also, harvested spleens and lymph nodes were mechanically disrupted and filtered using cell strainers to obtain single-cell suspensions. Red blood cells in the spleen cell suspensions were lysed using ACK (ammonium chloride-potassium) buffer; the remaining cells were washed and counted using a hemocytometer. The cells were stained with fluorochrome-conjugated antibodies for immune cell surface markers (anti-CD45, CD3, CD4, CD8, NK1.1, DX5, CD11c, F4/80, live/dead stain) as previously described (32) and acquired using a FACS Canto II.

### Intracellular Cytokine Staining

Single-cell suspensions obtained from the spleens, lymph nodes, and tumors were seeded in 24-well plates and stimulated with a cocktail containing phorbol myristate acetate (50 ng/ml), ionomycin (500 ng/ml), and brefeldin-A (10 µg/ml) for 4 h at 37°C. The cells were stained with antibodies for the surface markers CD3, CD4, and CD8 following the procedures described above. After surface staining, the cells were fixed with 4% paraformaldehyde for 15 min and washed with FACS buffer, then resuspended in saponin buffer (1 mg/ml, Sigma-Aldrich) for 15 min to permeabilize the cells. For intracellular cytokine staining, the cells were incubated for 30 min with fluorochrome-conjugated antibodies against the following cytokines: IFN-*γ*, IL-4 and IL-10, and acquired using a FACS Canto II as previously described (32). Results were analyzed using the FlowJo software (TreeStar Inc, Ashland, OR).

### Clonogenic Metastasis Assay

Lung metastasis was assessed using the clonogenic metastasis assay as described by Pulaski and Ostrand-Rosenberg (43). Briefly, the lungs were harvested from tumor-bearing mice and digested with collagenase IV solution (1.5 mg/ml, Sigma) at 4°C for 1 h. The slurry was filtered using 40 μm cell strainers and cultured in complete DMEM containing 6-thioguanine (final concentration 60 μM) at 37°C for 10–14 days when the metastatic 4T1 colonies became visible. The colonies formed in each culture dish were fixed with methanol, stained with 0.3% methylene blue and counted.

### Real-Time PCR Arrays

PCR array analysis was performed using a mouse tumor metastasis RT^2^ profiler array containing 84 key metastasis-related genes (Qiagen Corporation, Mississauga, ON, Canada; cat# PAMM-028Z). RNA was extracted from cell samples (4T1-EV and 4T1-PIP) using TRIzol (Thermofisher Scientific) and quantified using NanoDrop spectrophotometer (Thermofisher Scientific). The RT^2^ First Strand Kit (Qiagen Corporation) was used to reverse transcribe equal amounts of RNA, according to manufacturer’s suggested protocols. The cDNA samples were applied to each real-time PCR reaction on the mouse tumor metastasis RT^2^ Profiler PCR array. Real time PCR was carried out using the CFX 96 qPCR system (BioRad Laboratories). The cycle profile consisted of denaturation at 95°C for 10 min, followed by 40 cycles of 95°C for 15 s and 60°C for 1 min. Data were analyzed using the web-based PCR Array Data Analysis Software (Qiagen Corporation).

### qPCR

RNA was reverse transcribed using the Lunascript cDNA synthesis kit (New England BioLabs Inc., Whitby, ON, Canada) according to the manufacturer’s suggested protocols. For assays on the CFX 96 qPCR system, the cDNAs were then mixed with forward and reverse primers, nuclease free water and the PerfeCTa SYBR Green Fast Mix (Quantabio, Beverly, MA), following instructions from the manufacturer. Thermal cycling profile consisted of polymerase activation and DNA denaturation at 98°C for 30 s, followed by 40 cycles of 95°C for 15 s and 60°C for 60 s. Cycle threshold and relative quantitation values were calculated automatically by the CFX96 instrument software. Data were analyzed using the delta-delta CT method and presented as fold increase in gene expression levels relative to the housekeeping gene, GAPDH. Primer sequences for validated genes are shown in **Table 1**.

**Table 1:** Primer sequences used in validation of PCR array.

Gene	Forward 5′–3′	Reverse 5′–3′
MMP13	GATGACCTGTCTGAGGAAGACC	GCATTTCTCGGAGCCTGTCAAC
MMP3	CTCTGGAACCTGAGACATCACC	AGGAGTCCTGAGAGATTTGCGC
CCL7	CAGAAGGATCACCAGTAGTCGG	ATAGCCTCCTCGACCCACTTCT
GAPDH	CATCACTGCCACCCAGAAGACTG	ATGCCAGTGAGCTTCCCGTTCAG

### Statistical Analysis

Results are shown as mean ± SEM. Two-tailed Student’s t-test, Mann–Whitney test or ANOVA were used to compare the means from different groups of cells or mice. p ≤0.05 was considered significant.

## Results

### Generation of Prolactin Inducible Protein Expressing Mouse Breast Cancer Cells

**Figure 1:**
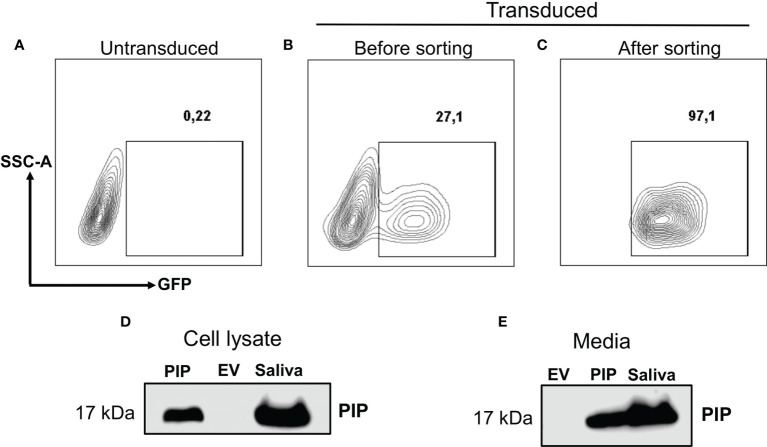
Generation and confirmation of PIP-expressing 4T1 cells. Lentivirus encoding the mouse *PIP* cDNA–*eGFP* construct was incubated with 4T1 cells for overnight incubation. Contour plots show the percentages of GFP expressing 4T1 cells for un-transduced cells **(A)** and transduced cells before **(B)** and after sorting **(C)** by flow cytometry. Western blot analysis using polyclonal rabbit anti-mouse PIP antibody was performed on 4T1 cell lysate **(D)** and cell culture media **(E)** as described in the *Materials and Methods* section. Mouse saliva was used as a positive control for PIP.

The 4T1 mouse BC cell line mimics the human stage 4 TNBC (43). As such, the transplantable syngeneic immunocompetent 4T1-BALB/c mouse model has been extensively used in preclinical studies (43). There are indeed many similarities between human and mice regarding tissue specific expression, although in the mouse, PIP is not expressed in the breast, perhaps attributed to species differences (20, 31, 45). Thus, 4T1 mouse BC cells that stably express PIP were generated. Lentiviral transduction was the strategy of choice as this method is known to induce a more stable gene expression after integrating the gene of interest into the genome of the host cell (46). The transduction efficiency was assessed by measuring the percentage of GFP expressing cells using flow cytometry. Subsequently, the transduced 4T1 cells were also sorted to high purity (>97%) by flow cytometry (**Figures 1A–C**).

The level of PIP produced by the transduced 4T1 cells was then assessed. Cell lysates were prepared and the Bicinchoninic acid (BCA) assay was used to determine the protein concentrations in the cell lysates before Western blot analysis. The PIP band was confirmed in the lysate of PIP-transduced 4T1 cells (4T1-PIP) but not in the lysate of the empty vector (4T1-EV) control cells (**Figure 1D**). Mouse saliva, which contains high levels of PIP, was used as a positive control. Since PIP is a secreted protein, we also collected cell culture supernatant and assessed for the presence of PIP by Western blot analysis. As shown in **Figure 1E**, PIP was detected in the media collected from 4T1-PIP cells but not in the media collected from 4T1-EV cells, confirming the successful transduction of 4T1 cells with the PIP-expressing vector and production of PIP by these 4T1 cells. For comparison with the 4T1 cells, we utilized the E0771 cell line, which is a medullary mammary carcinoma cell line originally isolated from a spontaneous tumor and is syngeneic to C57BL/6 mice (47). PIP-expressing E0771 cells were also generated by lentiviral transduction and Western blot analysis was employed as well to detect PIP levels in the cells (**Figures S1A–D**).

### Prolactin Inducible Protein Expression Does Not Alter the Characteristics of 4T1 and E0771 Cells *In Vitro*

**Figure 2:**
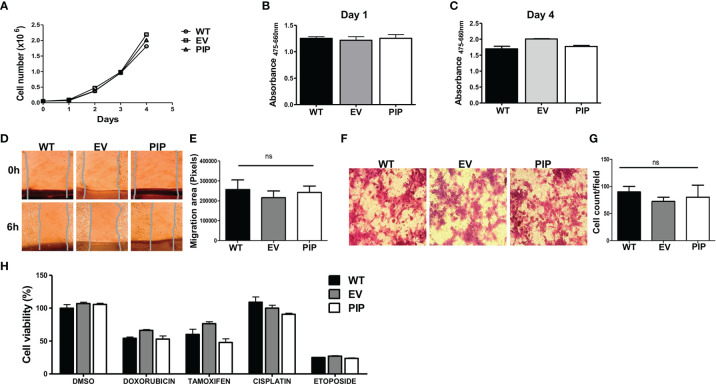
PIP expression does not affect 4T1 cell proliferation, migration, and sensitivity to chemotherapeutic agents *in vitro*. Wild-type (WT), empty vector (EV) or PIP transduced (PIP) 4T1 cells were seeded in 12 well plates (5 × 10^4^ cells/well) and counted daily for 4 days using the TC-10 cell counter. Panel **(A)** shows the growth curve for WT, EV and PIP transduced 4T1 cells. Panels **(B)** and **(C)** show the absorbance values for the different 4T1 cell lines at 1 and 4 days respectively, as assessed by the XTT assay. Cell migration was assessed by wound healing and trans-well migration assays. **(D)** Representative microscopic images of the wound areas at times 0 and 6 h post scratching. **(E)** Bar graph depicting the migration areas of the different 4T1 cell lines at 6 h post scratching (n = 4). **(F)** Representative images of 4T1 cell lines from the trans-well migration assay. **(G)** bar graph showing the number of migrated cell count per field (n = 3). WT, EV, and PIP transduced 4T1 cells were treated *in vitro* with different anticancer drugs (doxorubicin, cisplatin, etoposide) as well as tamoxifen for 48 h and cell viability was measured by XTT assay and normalized to DMSO control **(H)**. All experiments were done in triplicates, and the results are representative of three different experiments with similar outcomes. Results are expressed as mean± SEM; ns, not significant; two-way analysis of variance was conducted.

The effect of PIP expression on 4T1 cell proliferation was first assessed by utilizing the trypan blue dye exclusion assay (see *Material and Methods*). The 4T1-PIP cells and controls (4T1-EV and 4T1-WT) were grown for 4 days and counted daily using a Bio-Rad TC-10 counter. Results show that there was no difference in the proliferation rates between the 4T1-PIP cells, the 4T1-EV cells and 4T1-WT (**Figure 2A**). An alternate approach, the XTT assay, was also employed to measure 4T1 cell proliferation in the presence or absence of PIP expression. As shown in **Figures 2B, C**, and consistent with the cell count (**Figure 2A**), there was no difference in the proliferation rates of 4T1-PIP expressing cells and the corresponding controls. We also investigated the effect of PIP expression on apoptosis in 4T1 cells. We did not observe any significant differences in the expression of apoptosis and necrosis markers between empty vector and PIP-expressing 4T1 cells following Annexin-v and 7AAD staining (data not shown).

The effect of PIP expression on migration of 4T1 cells was also examined *in vitro*. As with the proliferation studies, two different approaches were utilized. First, using a wound healing/scratch assay, we found no significant difference in the migration rates of 4T1-PIP cells when compared to controls, following 6 h of incubation (**Figures 2D, E**). Next, using a trans-well migration assay (which measures the migration of cells in response to a chemotactic agent (fetal bovine serum, FBS), there was no difference in the number of 4T1-PIP cells that migrated across the trans-well membrane in response to the FBS after 24 h incubation compared to controls (**Figures 2F, G**).

Next, we also assessed whether PIP could affect the sensitivity of 4T1 mouse BC cells to anticancer drugs *in vitro*. Cells (4T1-PIP, 4T1-EV and 4T1-WT) were treated with doxorubicin, cisplatin or etoposide for 48 h and the metabolic activity of the surviving cells was assessed using the XTT assay. There was no significant difference in the sensitivity of PIP expressing 4T1 cells and controls to the drugs tested (**Figure 2H**). As well, PIP did not affect the sensitivity of 4T1 cells to tamoxifen, a selective estrogen receptor modulator (SERM) frequently used to treat estrogen receptor positive BC patients (48).

These same functional assays were also conducted to assess the effect of PIP expression on E0771 cell proliferation, migration and sensitivity to anticancer agents and tamoxifen. In terms of these cellular characteristics, as observed in the 4T1 cell line, there was no difference between PIP-expressing E0771 cells (E0771-PIP), empty vector control E0771 cells (E0771-EV) and wild-type E0771 cells (E0771-WT) (**Figures S2A–F**).

### Expression of Prolactin Inducible Protein Leads to Delayed Tumor Onset, Reduced Tumor Growth and Smaller Tumor Size *In Vivo*

**Figure 3:**
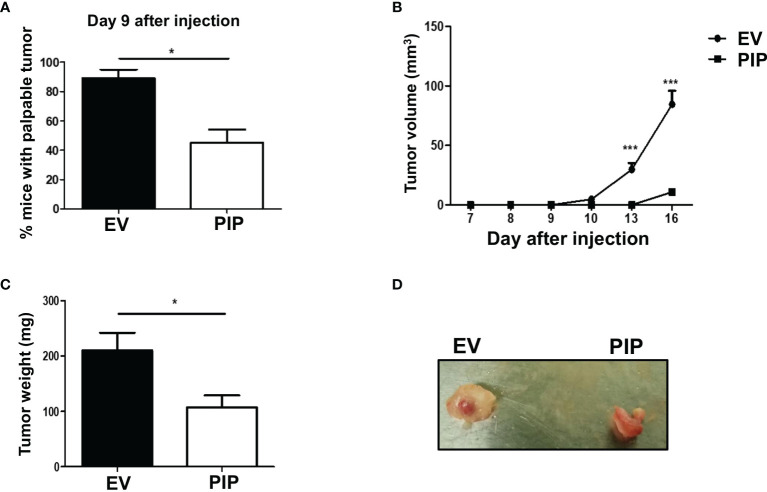
PIP expression delays 4T1 tumor onset and growth in immunocompetent mice. Female WT BALB/c mice were injected orthotopically into the 4^th^ mammary fat pad with 1 × 10^4^ 4T1-EV (empty vector control) or 4T1-PIP (PIP expressing) cells in 100 μl PBS and the onset of palpable tumor (latency) was determined **(A)**. Thereafter, the growth curves were determined by measuring the tumor diameter daily with digital calipers **(B)**. At sacrifice, the tumors were excised and weighed to determine the tumor size **(C)** and representative photo images of excised tumors were obtained **(D)**. Results are representative of three different experiments. Results are expressed as mean ± SEM of n = 3–5 mice/group. *p < 0.05; ***p < 0.001; T-test and two-way ANOVA were conducted.

The 4T1 syngeneic transplantable mouse model, a well-studied mouse BC model, mimics human stage IV BC (43). At this stage of BC, patient prognosis is often very poor and effective treatment options are limited due in part to poor understanding of the molecular mechanisms of the disease. Furthermore, the 4T1 model is a model of human TNBC, which has been reported to be relatively more immunogenic compared to other subtypes of BC and thus more amenable to immunomodulation (49). As well, 4T1 cells are capable of spontaneous metastasis to several distant organs such as the lungs, and are resistant to the drug 6-thioguanine, making it possible to detect micro-metastases in distant organs more accurately than in most other BC models (43). Thus, the 4T1 syngeneic transplantable mouse model was chosen to investigate the role of PIP in breast tumorigenesis, immune response and metastasis *in vivo*. Tumor latency, growth, and size were assessed following orthotopic injection of 4T1-PIP and 4T1-EV cells into the 4^th^ mammary fat pad of immunocompetent BALB/c mice. We found that tumor onset was delayed in the 4T1-PIP group compared to the 4T1-EV control group. Approximately 40% of the mice in the PIP group developed palpable tumors by 9 days after injection (**Figure 3A**), compared to more than 80% of mice in the control group. As well, there was a significant reduction in tumor growth and weight in the mouse group injected with 4T1-PIP cells when compared to the control group (**Figures 3B–D**). Furthermore, we also investigated the effect of PIP on breast tumor growth using another mouse BC cell line (E0771 cells). Interestingly, preliminary *in vivo* data with E0771 cells injected into C57BL/6 mice also showed that tumor growth and weight are reduced in mice bearing E0771-PIP tumors (**Figure S3**). This data validates our *in vivo* 4T1 studies and suggests that PIP may also reduce tumor growth in a different tumor line and on a different genetic background.

### Prolactin Inducible Protein Expression in 4T1 Tumors Alters the Composition of Immune Cells Within the Tumor Microenvironment

**Figure 4:**
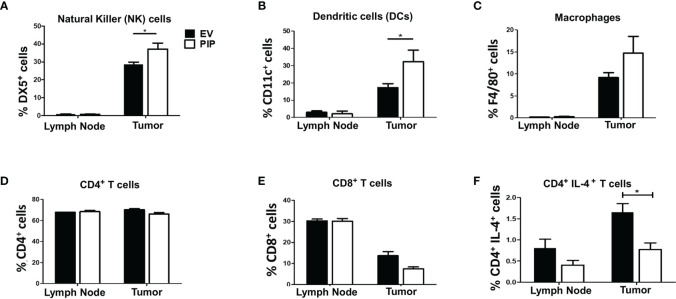
Expression of PIP gene by 4T1 tumor cells is associated with increased frequency of natural killer (NK) and dendritic cells and decreased CD4^+^IL-4^+^ T cells in tumor infiltrating cells. BALB/c mice were injected with PIP expressing 4T1 cells (PIP) and controls (EV) and after 14 days, mice were sacrificed and the percentage of NK cells (DX5^+^), **(A)**, dendritic cells (CD11c^+^), **(B)**, macrophages (F4/80^+^), **(C)**, CD4^+^ **(D)** and CD8^+^ **(E)** T cells, in the draining lymph nodes and tumors were assessed by flow cytometry. Some cells were stained directly *ex vivo*, and the frequency of IL-4-secreting CD4^+^ T cells was assessed by flow cytometry **(F)**. Results are representative of three different experiments. Results are expressed as mean ± SEM of n = 4–5 mice/group. *p < 0.05; two-way ANOVA was conducted.

Given that the immune response plays a critical role in suppressing tumor growth, we investigated the impact of PIP expression on the frequency of immune cells in the draining lymph nodes and tumor infiltrating lymphocytes (TIL) directly *ex vivo*. The draining lymph nodes and tumors from tumor bearing mice were collected, stained for immune cell markers and cytokines, and assessed by flow cytometry. We observed a significantly higher percentage of NK cells (**Figures 4A** and **S4A**) and DCs (**Figures 4B** and **S4B**) in the 4T1-PIP tumors. However, there was no significant difference in the percentages of macrophages, CD4^+^, and CD8^+^ T cells in the draining lymph nodes and tumors from mice injected with either 4T1-PIP or 4T1-EV cells (**Figures 4C–E**; **S4C, D**).

Cytokines have been reported to modulate antitumor immune responses, thereby playing either pro-tumorigenic or antitumorigenic roles depending on the type and quantity of the cytokine (50). Therefore, the impact of PIP expression on cytokine responses in the draining lymph nodes and tumors from mice bearing 4T1-PIP tumors and 4T1-EV tumors was assessed. Although there was no difference in the lymph nodes, there was a significant decrease in numbers of IL-4-producing CD4^+^ T cells in PIP expressing 4T1 tumors compared to controls (**Figures 4F** and **S4E**). Interestingly, there was no significant difference in the frequency of IFN-*γ*-producing CD4^+^ T cells in either the tumor or the draining lymph nodes (**Figure S5**) or in the spleens (**Figure S6**). As well, we did not observe any difference in the ratio of IFN-*γ* to IL-4 production in the 4T1-PIP and 4T1-EV tumor cells as assessed by enzyme-linked immunosorbent assay, ELISA (**Figure S4F**).

### The Delayed Onset, Reduced Growth, and Size of 4T1-PIP Tumors in Immunocompetent BALB/c Mice Is Abrogated in Immunodeficient BALB/c Mice

**Figure 5:**
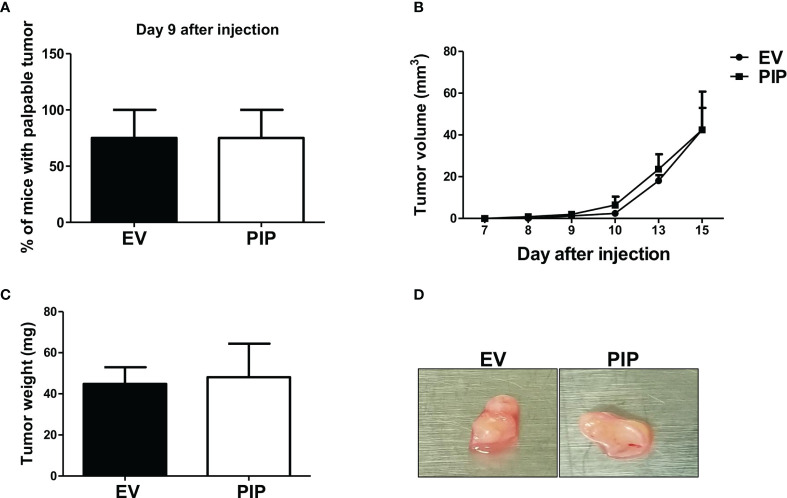
Delayed onset and reduced tumor growth by 4T1-PIP cells is abrogated in immunodeficient mice. PIP expressing 4T1 tumors (PIP) and empty vector controls (EV) were injected into Rag2^−/−^*γ*c^−/−^ double knockout (DKO) BALB/c mice and onset of palpable tumor (latency) was determined **(A)**. The tumor growth was measured with digital calipers **(B)**. At sacrifice, the tumors were excised, and the weight was determined **(C)**. A representative photo image of the excised tumors is shown **(D)**. Results are representative of two different experiments. Results are expressed as mean ± SEM of n = 4 mice/group, T-test and two-way ANOVA were conducted.

To determine whether the observed reduction in tumor size and growth observed in mice injected with 4T1-PIP cells is mediated by immune cells, 4T1-PIP and 4T1-EV cells were injected orthotopically into the 4^th^ mammary fat pad of the Rag2/common gamma chain deficient (Rag2^−/−^ *γ*c^−/−^), a mouse line which lack NK, T and B cells. In contrast to our previous findings in immunocompetent BALB/c mice (**Figure 3**), here, no difference in any of these parameters (latency, growth, size; **Figures 5A–D**) between 4T1-PIP and 4T1-EV tumors grown in immunodeficient BALB/c mice was observed.

### PIP Expression Is Associated With Increased Metastasis to the Lungs in Both Immunocompetent and Immunodeficient BALB/c Mice

**Figure 6:**
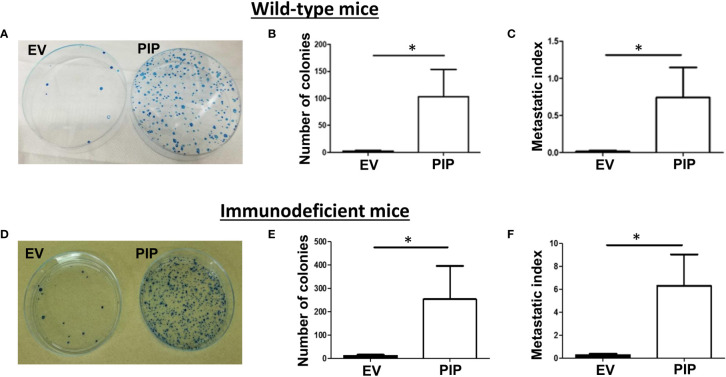
PIP expression by 4T1 cells is associated with increased lung metastasis. Wild-type (WT) and Rag2^−/−^γc^−/−^ double knockout (DKO) female BALB/c mice were injected with PIP expressing (PIP) or empty vector control (EV) 4T1 cells and after 14 days, mice were sacrificed, and lung metastasis was assessed by the clonogenic metastasis assay. Panels show representative images of the 4T1 colonies **(A, D)**, the number of metastatic 4T1 colonies **(B, E)** and the metastatic index **(C, F)**, respectively, for WT **(A–C)** and DKO **(D–F)** mice. Results representative of two to three experiments and are expressed as mean ± SEM. n = 3–5 mice per group. *p < 0.05; Mann–Whitney test was conducted.

The 4T1 mouse BC cells are known to metastasize to distant organs including the lungs (43). Therefore, at the experimental endpoint, lungs were collected from tumor bearing mice and assessed for lung metastasis by the clonogenic metastasis assay (see *Materials and Methods*). Interestingly, a higher number of lung metastatic 4T1-PIP colonies were observed in both WT (**Figures 6A, B**) and immunodeficient mice (**Figures 6D, E**), compared to 4T1-EV controls. We also observed that the metastatic index (the number of metastatic colonies divided by the tumor size), was significantly higher in mice bearing 4T1-PIP tumors compared to mice bearing 4T1-EV tumors (**Figures 6C, F**). Furthermore, we isolated 4T1-PIP and 4T1-EV cells from the primary tumor site and metastatic foci and confirmed by Western blot analysis that the 4T1-PIP cells at these sites retain PIP expression (data not shown).

### Prolactin Inducible Protein Expression Leads to Increased Expression of Chemokine Ligand 7 and Matrix Metalloproteinases 3 and 13

**Figure 7:**
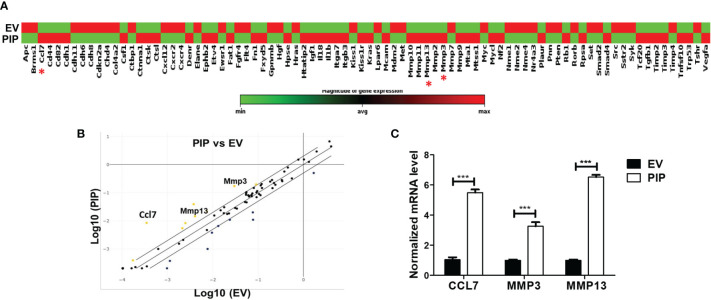
PIP expressing 4T1 cells upregulate the expression of CCL7, MMP3, and MMP13. A qPCR array analysis of mouse tumor metastasis genes was performed in 4T1-PIP and 4T1-EV cells prior to injection into mice (denoted as PIP and EV). **(A)** shows the gene expression profile of 4T1-PIP and 4T1-EV cells, with CCL7, MMP3 and MMP13 marked with red asterisk. The data in **(A)** represents single samples. **(B)** is a scatter plot representing the relative expression of metastasis genes by 4T1-PIP cells compared to 4T1-EV cells with a threshold of two-fold upregulation (yellow dots) or down regulation (dark blue dots). The genes for CCL7, MMP3 and MMP13 (metastasis-related genes) showed consistent upregulation in 4T1-PIP cells and were selected for further validation by qPCR. **(C)** shows the normalized (relative to GAPDH) fold expression levels of MMP3, MMP13 and CCL7 in 4T1-PIP and 4T1-EV cells. Data are representative of two experiments and are shown as mean ± SEM. ***p < 0.001; two-way ANOVA was conducted.

To gain insight into how the expression of PIP in 4T1 cells might enhance metastasis to the lungs, we conducted qPCR array analysis to investigate whether PIP alters the expression of metastasis related genes in the 4T1 cells. Using an RT^2^ PCR array, we assessed the expression of genes related to mouse tumor metastasis in 4T1-PIP cells and compared them with 4T1-EV cells. Out of the 84 genes analyzed, CCL7 as well as two matrix metalloproteinase genes, MMP3 and MMP13, were consistently found to be significantly upregulated in 4T1-PIP cells (**Figures 7A, B**). The upregulation of these genes were further validated using qPCR analysis (**Figure 7C**).

## Discussion

Although PIP is highly expressed in some BCs, its role in BC pathogenesis is not known. However, studies have shown that PIP is a multifaceted protein which can regulate components of both the innate and adaptive immune response (31, 38). PIP is located at sites considered to be the first line of defense against pathogen entry (38, 39) and our group earlier demonstrated that PIP promotes oral bacterial aggregation thereby contributing to innate host defense (41). Using PIP KO mice, we also demonstrated that loss of PIP results in impaired DC function and impaired differentiation of naïve CD4^+^ T cells into IFN-*γ*-producing CD4^+^ T (Th1) cells (32). These findings provide evidence to suggest that PIP is important in T cell-mediated immunity, an arm of the adaptive immune response that is critical for antitumor immunity in BC (51). As well, the expression of PIP in BC patients has been associated with better prognosis and response to chemotherapy (27–30), suggesting that PIP may be protective against BC. Therefore, this study was undertaken to assess the possible role of PIP in BC and to investigate whether PIP enhances antitumor immune responses in BC and in so doing, confers protection against BC.

We expressed PIP in 4T1 cells and conducted functional assays to determine whether PIP expression in 4T1 cells alters important cancer cell characteristics including proliferation and migration. To evaluate whether the effect of PIP on 4T1 cell characteristics can be reproduced in another mouse BC cell line, we also expressed PIP in the E0771 cell line and conducted the same functional assays. We did not observe any changes in 4T1 or E0771 cell proliferation and migration *in vitro* following PIP expression suggesting that PIP does not affect these characteristics in both 4T1 and E0771 cells. There have been contrasting reports in the literature concerning the effect of PIP on BC cell proliferation and migration *in vitro*. While some studies suggest that PIP increases cell proliferation (25, 52) and migration (53), others suggest that PIP is associated with reduced cell proliferation (54, 55) and migration (54). It is difficult to compare these studies due to different cell lines and experimental approaches employed. For instance, in these proliferation and migration studies, PIP was often either added to the media or knocked down in the BC cells, whereas in our study, PIP was stably expressed in the BC cells. Another difference is that these reports utilized human BC cell lines (such as T47D, MCF7 and MDA-MB231 cells) while mouse BC cell lines (4T1 and E0771 cells) were employed in our study. Thus, it appears that the effect of PIP on BC cell proliferation and migration *in vitro* depends on the cell line under investigation and/or experimental conditions utilized.

We showed that in immunocompetent BALB/c mice, 4T1-PIP tumors exhibited delayed tumor onset and reduced tumor growth compared to 4T1-EV control tumors unlike the *in vitro* studies where there was no difference in the proliferation of 4T1-PIP and 4T1-EV cells. This was also observed in the E0771-C57BL/6 mouse BC model. Thus, these *in vivo* studies suggested to us that the effect of PIP expression on 4T1 primary tumor growth was indirect. We also observed higher percentages of NK cells and DCs as well as lower percentages of Th2 cells in the 4T1-PIP tumors. NK cells and DCs can promote antitumor immune responses while Th2 cells are pro-tumorigenic (51). Thus, these findings suggest that PIP expression was altering the antitumor immune response.

To further confirm that PIP was affecting tumor growth *via* an immune response, we injected PIP expressing 4T1 cells into the mammary fat pads of immunodeficient (*Rag2*^−/−^*γc*^−/−^) mice which lack NK, T, and B cells. This was critical to determine whether enhanced antitumor immune activity is responsible for the reduced tumor growth observed in immunocompetent WT mice bearing 4T1-PIP tumors. When the tumors developed in these immunodeficient mice, we saw no difference in tumor onset and tumor growth in 4T1-PIP compared to 4T1-EV control tumors. These studies suggest that the delayed onset and slow growth of 4T1-PIP primary tumors in WT mice are regulated at least in part by immune cells. In addition to the now accumulating body of evidence to suggest an important role for PIP in the immune response such as its ability to interact with the CD4 molecule of T cells, and immunoglobulins produced by B cells (38), we also demonstrated a direct effect of PIP on T cells, DCs (32), and macrophages (33). Our current data now suggest that PIP may also modulate NK cell immune responses in BC. Furthermore, we demonstrated a direct effect of PIP on T cells, DCs (32), and macrophages (33); these data suggest that PIP may also modulate NK cell immune responses in BC. Further studies are warranted to determine the exact immune cell type(s) responsible for this effect of PIP in 4T1 tumors and the mechanisms involved. One such approach would be to utilize the immunodeficient BALB/c mice as a “reconstitution” model with highly enriched immune cell subtype, for example NK or CD3^+^ (T) cells.

We also evaluated the effect of PIP expression on the sensitivity of 4T1 and E0771 cells to chemotherapeutic agents (doxorubicin, cisplatin, etoposide) used in the treatment of BC and observed that PIP expression does not directly affect the sensitivity of both cell lines to these drugs *in vitro*. As well, PIP did not affect the sensitivity of the cells to tamoxifen, a selective estrogen receptor modulator used for the treatment of BC patients. These studies were prompted by a recent report that patients with PIP-expressing tumors responded better to chemotherapy (doxorubicin) (30). These *in vitro* results therefore suggest that PIP may be exerting an indirect effect on the sensitivity of BC cells to chemotherapeutic agents. Indeed, immune cells have been reported to synergize with chemotherapeutic agents to facilitate tumor eradication (56). It is thus plausible that the positive response to chemotherapy observed in BC patients with PIP-expressing tumors may be immune related as this important component (immune system) was absent in our *in vitro* drug sensitivity assay.

As previously mentioned, 4T1 cells generally metastasize to the lungs. We therefore evaluated whether PIP affected the metastatic rate in the lungs. Interestingly, we observed that PIP expression was associated with increased numbers of 4T1 colonies in the lungs in both immunocompetent and immunodeficient mice. Reduced primary tumor growth coupled with increased lung metastasis would also suggest that PIP increased the metastatic potential of the 4T1 tumors. The early dissemination of BC cells has been reported to contribute to higher metastasis (57, 58). When we assessed metastasis at an earlier time point (day 12), we observed higher metastasis in the PIP group, although the difference was found to be not statistically significant (data not shown). Therefore, it is plausible that early seeding/dissemination of the 4T1-PIP cells may lead to the increased lung metastasis and larger metastatic burden at endpoint. Collectively, these results suggest that in the case of BC, PIP may function as a double-edged sword, in one scenario enhancing local antitumor immune response, whereas in another promoting lung metastasis.

To gain further insight regarding the possible mechanisms by which PIP would enhance the metastatic potential of 4T1 cells, we examined the differences in the expression of metastasis related genes in 4T1-PIP and 4T1-EV control cells. Several differentially expressed metastatic genes were identified. However, three specific genes of interest, CCL7, MMP3, and MMP13 were consistently upregulated in the 4T1-PIP cells compared to 4T1-EV controls (**Figure 7**). CCL7 has been previously reported to be expressed in BC, but its role in BC is not well known (59). In general, CCL7 has been shown to play dual roles in cancer progression (60) by promoting the recruitment of immune cells such as NK cells, DCs, and macrophages into the tumor microenvironment (61, 62) as well as cancer metastasis (63–65). Given that the expression of PIP in 4T1 cells also led to an increased infiltration of NK cells and DCs (**Figure 4**) and higher metastasis (**Figure 6**), it is plausible that an upregulation of CCL7 in 4T1-PIP cells may lead to enhanced immune cell infiltration of 4T1-PIP tumors (thereby reducing primary tumor growth) as well as increased lung metastasis. MMP3 and MMP13 are matrix metalloproteinases which degrade extracellular matrix proteins and can also facilitate cancer cell metastasis to the lungs (66). It is conceivable that PIP may upregulate the expression of CCL7, MMP3 and MMP13 by regulating pathways that induce the expression of these genes. We have shown in a previous report that PIP regulates the mitogen-activated protein kinase (MAPK) and signal transducer of activation of transcription (STAT) pathways (33), and these pathways are among those involved in the expression of MMPs (67).

The role of PIP in metastasis may also be related to its ability to degrade fibronectin due to its inherent aspartyl protease activity (68). Fibronectin is a component of the extracellular matrix, and studies have shown that it is important in regulating tumor cohesion (69), and increase in fibronectin has been linked to reduced metastasis (70). As a result, when fibronectin is disrupted, this could lead to loss of tumor integrity resulting in tumor cell detachment and promotion of metastasis. Of note, a recent study has also shown that fragments from fibronectin degradation can induce the expression of MMP3 and MMP13 in chondrocytes through the Myd88-dependent toll like receptor 2 (TLR2) signaling pathway (71). Although this was demonstrated in an arthritis model, it is possible that this mechanism is used in different disease states including BC such that fibronectin fragments generated by PIP activity may also upregulate the expression of MMP3 and MMP13 in the 4T1-PIP tumors. Yet another potential mechanism by which PIP enhances metastasis is by influencing the lung environment in a manner that favors metastatic colonization by the 4T1 cells. In a similar study it was shown that 4T1 cells in the primary tumor can enhance metastasis in the lungs by inducing the expression of TARC/CCL17 and MDC/CCL22 genes (72).

In conclusion, we have made a novel and important observation that PIP, a clinically relevant BC biomarker, can suppress primary tumor growth and also enhance metastasis during BC progression. A better understanding of the molecular events that occur between PIP and the tumor microenvironment has the potential to guide the development of novel therapeutic strategies that will benefit many BC patients.

## Data Availability Statement

The original contributions presented in the study are included in the article/**Supplementary Material**. Further inquiries can be directed to the corresponding authors.

## Ethics Statement

The animal study was reviewed and approved by University of Manitoba Animal Care Committee.

## Author Contributions

CE, SK, JU, and YM conceptualized the study. JU and YM acquired the funding. CE, NI, GA, LT, MM, ES, and AB investigated the study. CE, NI, GA, LT, MM, ES, AB, JU, and YM developed the methodology. Project administration was made CE, JU, and YM. JU and YM provided the resources. JU and YM supervised the study. CE was in charge of the visualization and wrote the original draft. CE, NI, SK, JU, and YM wrote, reviewed, and edited the article. All authors contributed to the article and approved the submitted version.

## Funding

We acknowledge the grant support from CancerCare Manitoba Foundation (grant number:761038013) (YM and JU) and the Natural Sciences and Engineering Research Council of Canada, NSERC (grant number RGPIN/5539-2014) (YM).

## Conflict of Interest

The authors declare that the research was conducted in the absence of any commercial or financial relationships that could be construed as a potential conflict of interest.
