# Supplementary material for: The Prolactin Inducible Protein Modulates Antitumor Immune Responses and Metastasis in a Mouse Model of Triple Negative Breast Cancer
Source: Front Oncol. 2021 Mar 12;11:639859. doi: 10.3389/fonc.2021.639859 (PMC7994859; doi:10.3389/fonc.2021.639859)
Supplement: Supplementary file 1 [file Presentation_1.pptx]

## Slide 1
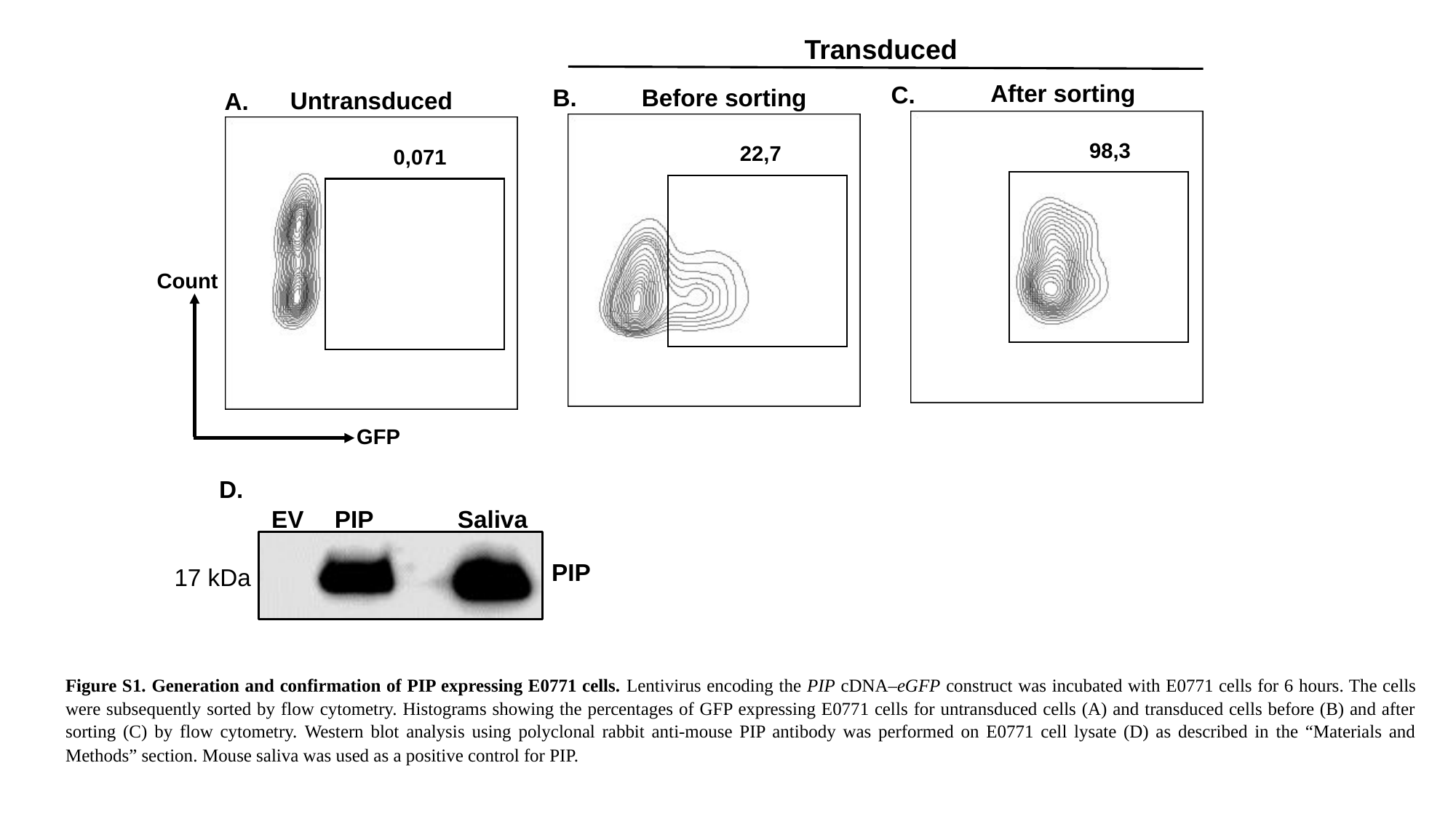

Transduced
After sorting
C.
B.
Before sorting
Untransduced
A.
98,3
22,7
0,071
Count
GFP
D.
EV
Saliva
PIP
PIP
17 kDa
Figure S1. Generation and confirmation of PIP expressing E0771 cells. Lentivirus encoding the PIP cDNA–eGFP construct was incubated with E0771 cells for 6 hours. The cells were subsequently sorted by flow cytometry. Histograms showing the percentages of GFP expressing E0771 cells for untransduced cells (A) and transduced cells before (B) and after sorting (C) by flow cytometry. Western blot analysis using polyclonal rabbit anti-mouse PIP antibody was performed on E0771 cell lysate (D) as described in the “Materials and Methods” section. Mouse saliva was used as a positive control for PIP.

## Slide 2
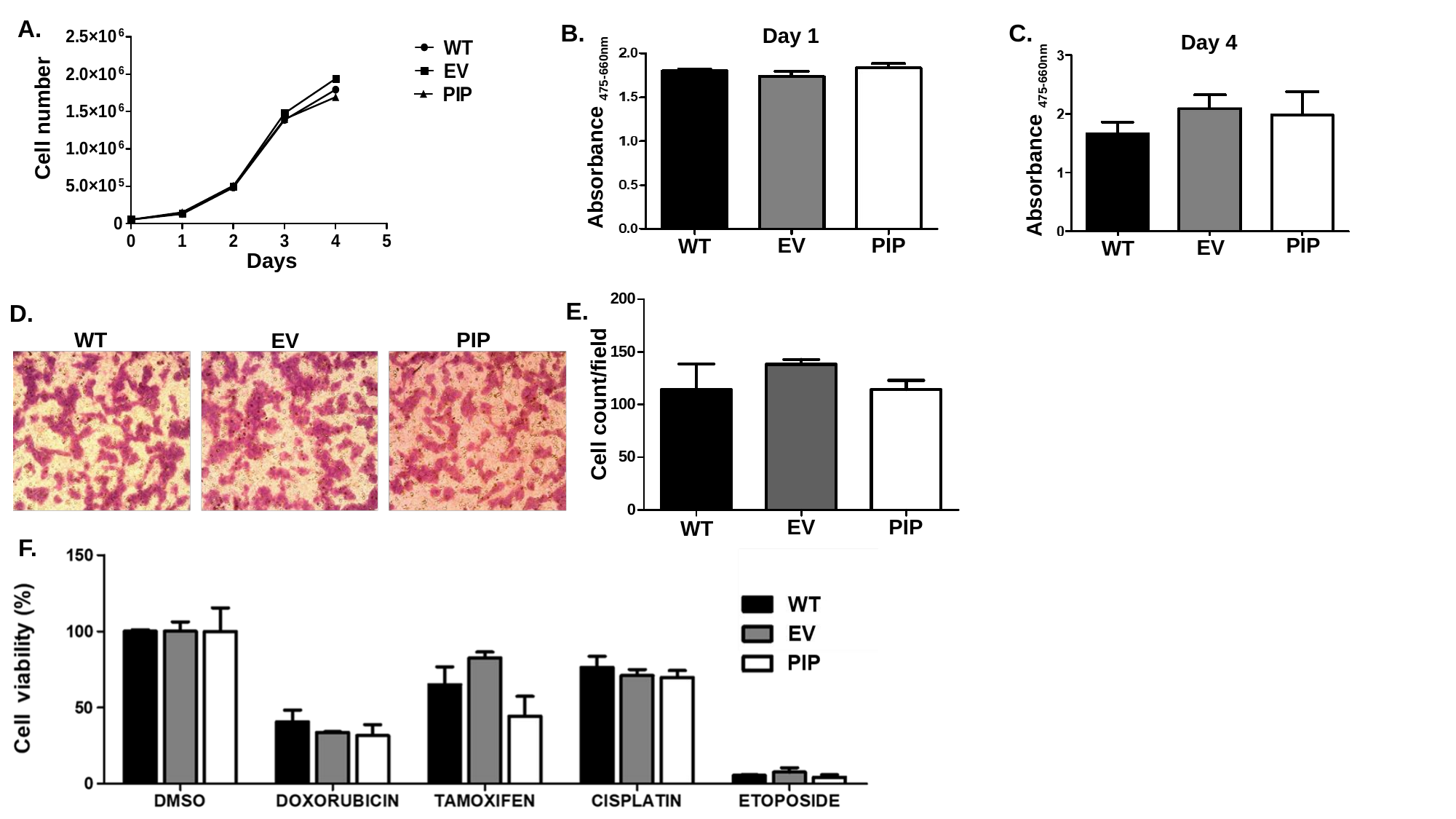

A.
B.
C.
Day 1
Day 4
Cell number
Absorbance 475-660nm
Absorbance 475-660nm
EV
PIP
PIP
WT
EV
WT
Days
E.
D.
PIP
WT
EV
Cell count/field
EV
PIP
WT
F.

## Slide 3
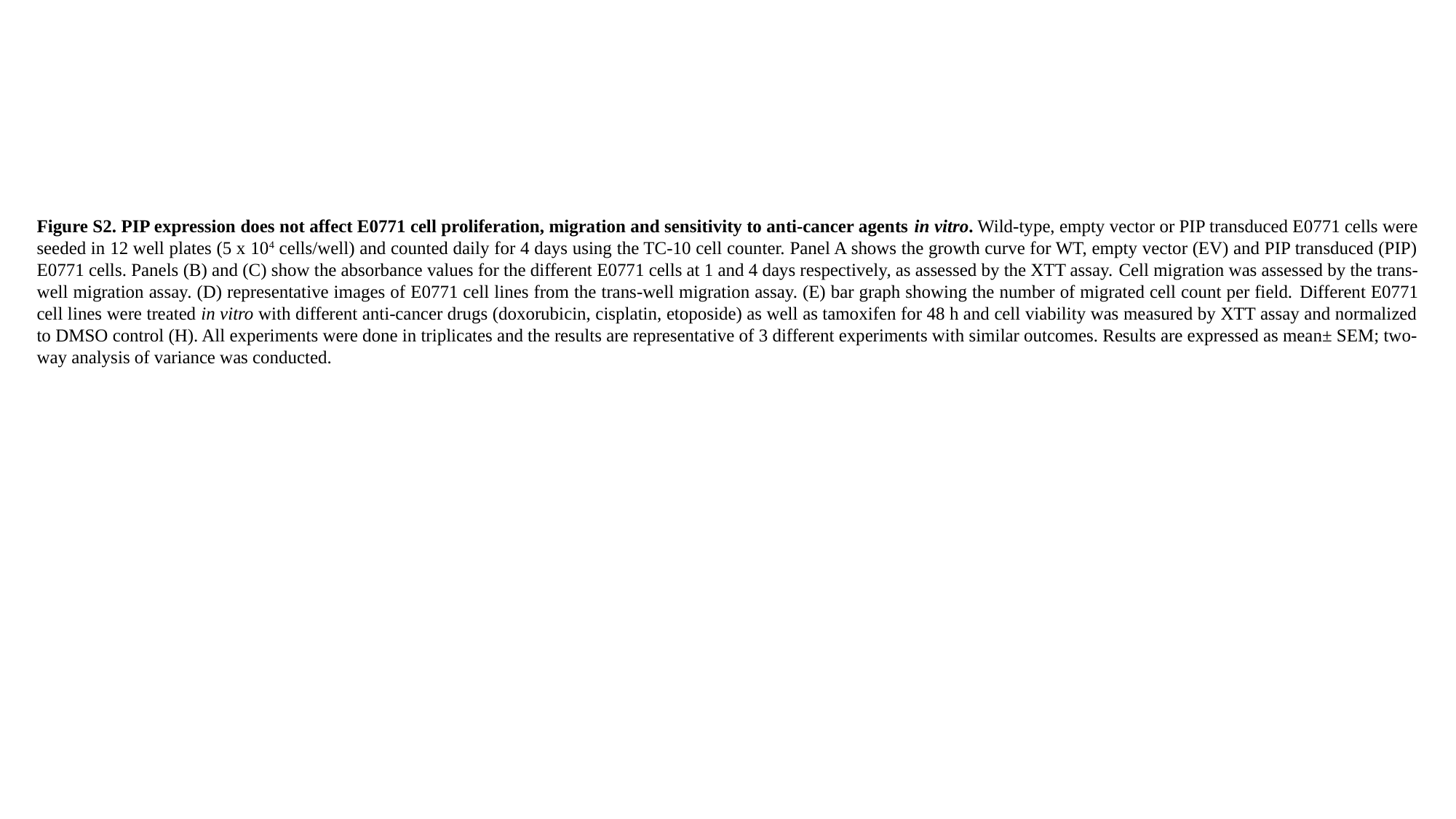

Figure S2. PIP expression does not affect E0771 cell proliferation, migration and sensitivity to anti-cancer agents in vitro. Wild-type, empty vector or PIP transduced E0771 cells were seeded in 12 well plates (5 x 10^4^ cells/well) and counted daily for 4 days using the TC-10 cell counter. Panel A shows the growth curve for WT, empty vector (EV) and PIP transduced (PIP) E0771 cells. Panels (B) and (C) show the absorbance values for the different E0771 cells at 1 and 4 days respectively, as assessed by the XTT assay. Cell migration was assessed by the trans-well migration assay. (D) representative images of E0771 cell lines from the trans-well migration assay. (E) bar graph showing the number of migrated cell count per field. Different E0771 cell lines were treated in vitro with different anti-cancer drugs (doxorubicin, cisplatin, etoposide) as well as tamoxifen for 48 h and cell viability was measured by XTT assay and normalized to DMSO control (H). All experiments were done in triplicates and the results are representative of 3 different experiments with similar outcomes. Results are expressed as mean± SEM; two-way analysis of variance was conducted.

## Slide 4
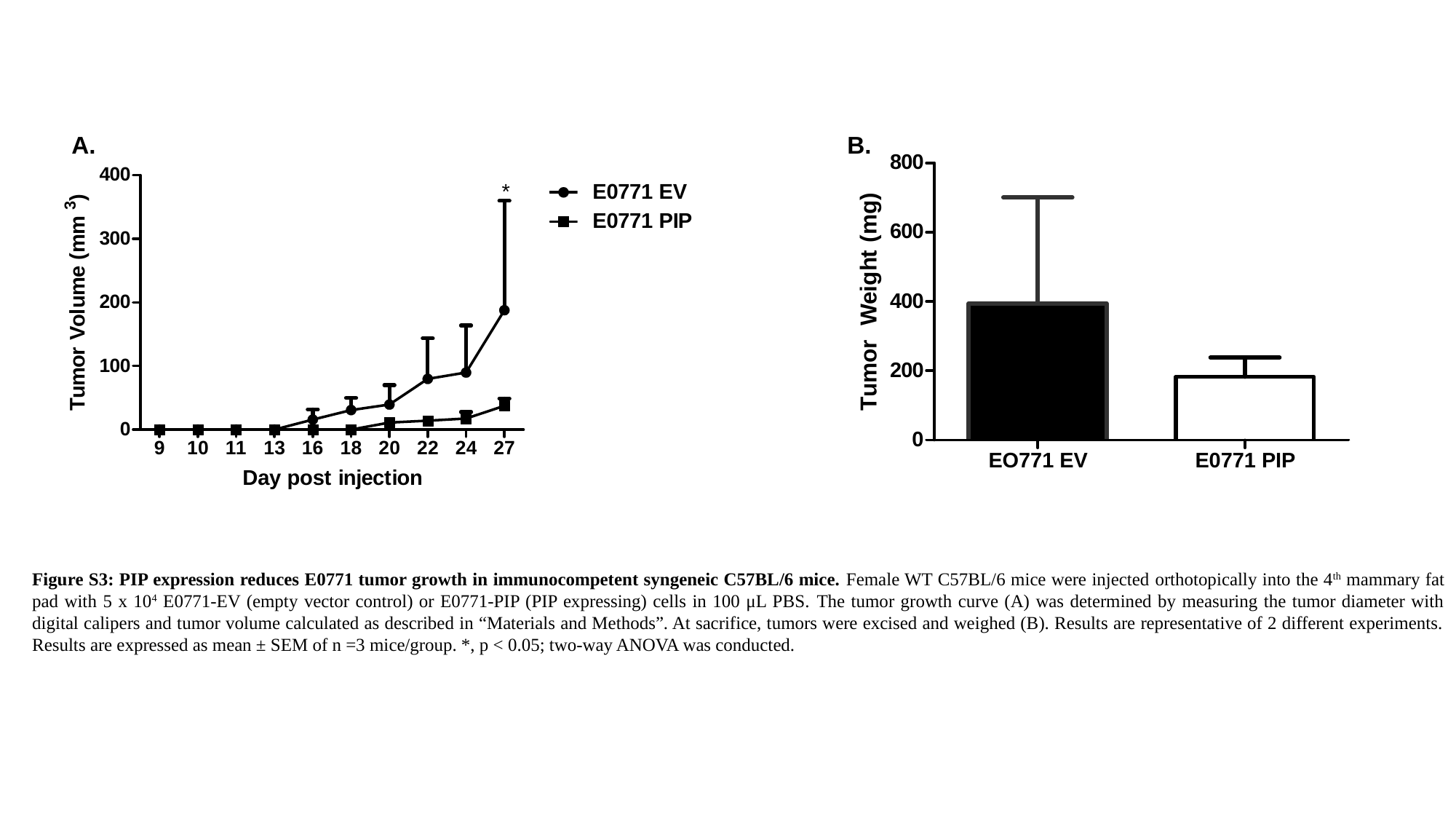

A.
B.
Figure S3: PIP expression reduces E0771 tumor growth in immunocompetent syngeneic C57BL/6 mice. Female WT C57BL/6 mice were injected orthotopically into the 4th mammary fat pad with 5 x 10^4^ E0771-EV (empty vector control) or E0771-PIP (PIP expressing) cells in 100 μL PBS. The tumor growth curve (A) was determined by measuring the tumor diameter with digital calipers and tumor volume calculated as described in “Materials and Methods”. At sacrifice, tumors were excised and weighed (B). Results are representative of 2 different experiments. Results are expressed as mean ± SEM of n =3 mice/group. *, p < 0.05; two-way ANOVA was conducted.

## Slide 5
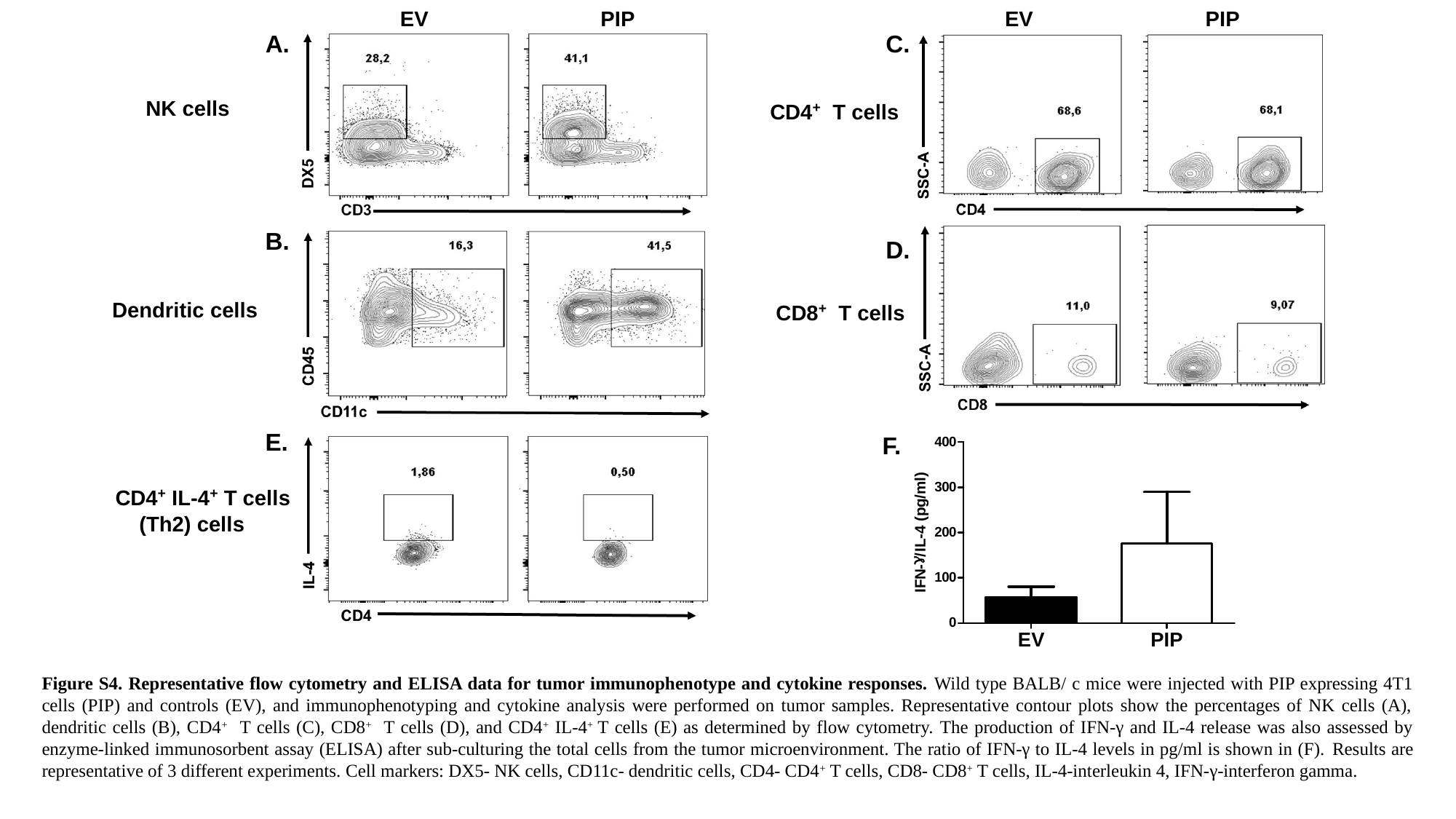

EV
PIP
EV
PIP
A.
C.
NK cells
CD4+ T cells
B.
D.
Dendritic cells
CD8+ T cells
E.
F.
CD4+ IL-4+ T cells (Th2) cells
Figure S4. Representative flow cytometry and ELISA data for tumor immunophenotype and cytokine responses. Wild type BALB/ c mice were injected with PIP expressing 4T1 cells (PIP) and controls (EV), and immunophenotyping and cytokine analysis were performed on tumor samples. Representative contour plots show the percentages of NK cells (A), dendritic cells (B), CD4+ T cells (C), CD8+ T cells (D), and CD4+ IL-4+ T cells (E) as determined by flow cytometry. The production of IFN-γ and IL-4 release was also assessed by enzyme-linked immunosorbent assay (ELISA) after sub-culturing the total cells from the tumor microenvironment. The ratio of IFN-γ to IL-4 levels in pg/ml is shown in (F). Results are representative of 3 different experiments. Cell markers: DX5- NK cells, CD11c- dendritic cells, CD4- CD4+ T cells, CD8- CD8+ T cells, IL-4-interleukin 4, IFN-γ-interferon gamma.

## Slide 6
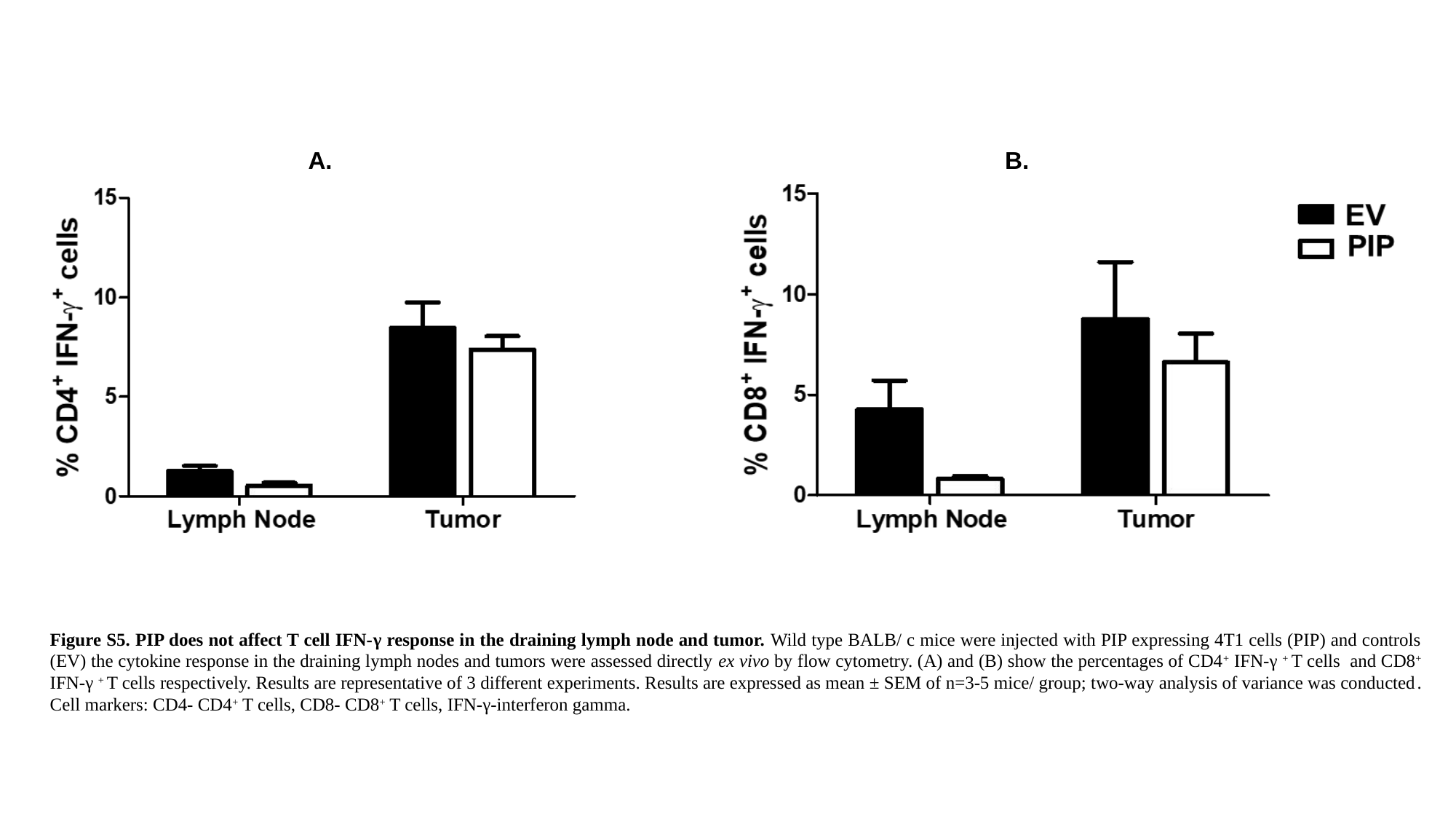

A.
B.
Figure S5. PIP does not affect T cell IFN-γ response in the draining lymph node and tumor. Wild type BALB/ c mice were injected with PIP expressing 4T1 cells (PIP) and controls (EV) the cytokine response in the draining lymph nodes and tumors were assessed directly ex vivo by flow cytometry. (A) and (B) show the percentages of CD4+ IFN-γ + T cells and CD8+ IFN-γ + T cells respectively. Results are representative of 3 different experiments. Results are expressed as mean ± SEM of n=3-5 mice/ group; two-way analysis of variance was conducted. Cell markers: CD4- CD4+ T cells, CD8- CD8+ T cells, IFN-γ-interferon gamma.

## Slide 7
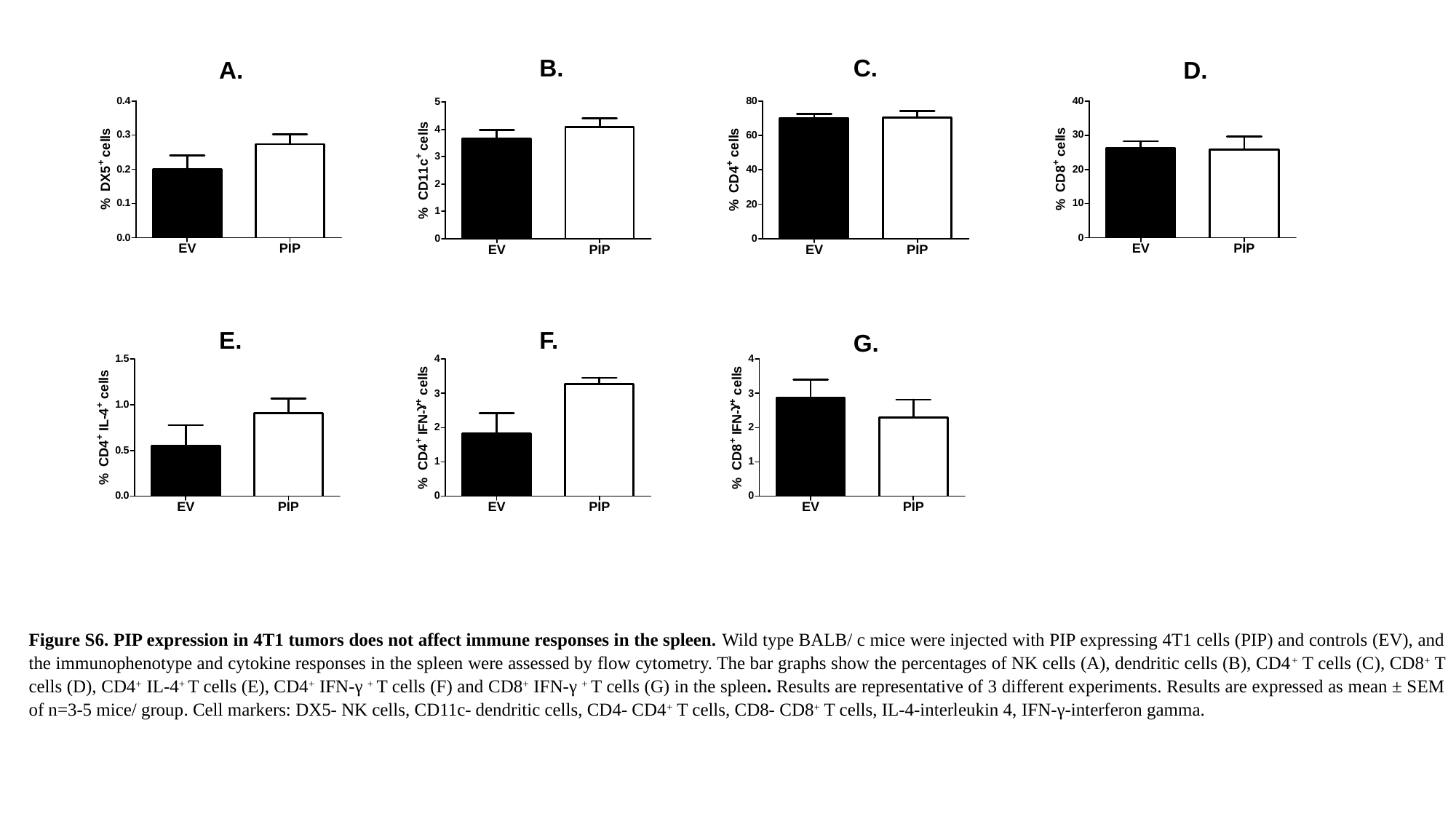

C.
B.
A.
D.
E.
F.
G.
Figure S6. PIP expression in 4T1 tumors does not affect immune responses in the spleen. Wild type BALB/ c mice were injected with PIP expressing 4T1 cells (PIP) and controls (EV), and the immunophenotype and cytokine responses in the spleen were assessed by flow cytometry. The bar graphs show the percentages of NK cells (A), dendritic cells (B), CD4+ T cells (C), CD8+ T cells (D), CD4+ IL-4+ T cells (E), CD4+ IFN-γ + T cells (F) and CD8+ IFN-γ + T cells (G) in the spleen. Results are representative of 3 different experiments. Results are expressed as mean ± SEM of n=3-5 mice/ group. Cell markers: DX5- NK cells, CD11c- dendritic cells, CD4- CD4+ T cells, CD8- CD8+ T cells, IL-4-interleukin 4, IFN-γ-interferon gamma.
